# NOD-Like Receptors: Guards of Cellular Homeostasis Perturbation during Infection

**DOI:** 10.3390/ijms22136714

**Published:** 2021-06-23

**Authors:** Gang Pei, Anca Dorhoi

**Affiliations:** 1 Institute of Immunology, Friedrich-Loeffler-Institut, 17493 Greifswald, Germany; 2 Faculty of Mathematics and Natural Sciences, University of Greifswald, 17489 Greifswald, Germany

**Keywords:** innate immunity, NOD-like receptors, pathogens, cellular homeostasis, NOD1/2, NLRP3, NLRP1

## Abstract

The innate immune system relies on families of pattern recognition receptors (PRRs) that detect distinct conserved molecular motifs from microbes to initiate antimicrobial responses. Activation of PRRs triggers a series of signaling cascades, leading to the release of pro-inflammatory cytokines, chemokines and antimicrobials, thereby contributing to the early host defense against microbes and regulating adaptive immunity. Additionally, PRRs can detect perturbation of cellular homeostasis caused by pathogens and fine-tune the immune responses. Among PRRs, nucleotide binding oligomerization domain (NOD)-like receptors (NLRs) have attracted particular interest in the context of cellular stress-induced inflammation during infection. Recently, mechanistic insights into the monitoring of cellular homeostasis perturbation by NLRs have been provided. We summarize the current knowledge about the disruption of cellular homeostasis by pathogens and focus on NLRs as innate immune sensors for its detection. We highlight the mechanisms employed by various pathogens to elicit cytoskeleton disruption, organelle stress as well as protein translation block, point out exemplary NLRs that guard cellular homeostasis during infection and introduce the concept of stress-associated molecular patterns (SAMPs). We postulate that integration of information about microbial patterns, danger signals, and SAMPs enables the innate immune system with adequate plasticity and precision in elaborating responses to microbes of variable virulence.

## 1. Introduction

Nucleotide binding oligomerization domain (NOD)-like receptors (NLRs) are a group of evolutionarily conserved pattern recognition receptors (PRRs) critical for microbial recognition and host defense. To date, 22 NLRs have been identified in humans and 34 distinct ones in mice. They have a common molecular organization comprising a subclass-specific N-terminal effector domain, a central NOD domain, and C-terminal leucine-rich repeats (LRRs) that bind to pathogen-associated molecular patterns (PAMPs). Based on the distinct N-terminal effector domains including transactivator domain (AD), baculovirus inhibitor repeats (BIRs), caspase recruitment domain (CARD), or pyrin domain (PYD), NLRs are divided into four subgroups: NLRA, NLRB, NLRC, and NLRP [1,2]. The Major Histocompatibility Complex Class II Transactivator (CIITA), the only member of NLRA, induces expression of the major histocompatibility complex (MHC) class I and II by functioning as a transcriptional activator and a transcription factor, respectively [3,4]. The NLRB subgroup contains only one single member in humans, the NLR family apoptosis inhibitory protein (NAIP), and seven members (NAIP1-7) in mice. NAIP and NAIP1 sense needle proteins of the type III secretion system (T3SS) and activate the NLRC4 inflammasome, while NAIP5/6 and NAIP2 recognize bacterial flagellin and rod components, respectively [5,6,7,8]. The NLRC subgroup includes NOD1 and NOD2, which are well-characterized PRRs recognizing bacterial peptidoglycan. They sense γ-d-glutamyl-meso-diaminopimelic acid (iE-DAP) and muramyl dipeptide (MDP), respectively [9,10,11,12]. Ligand engagement leads to the release of auto-inhibitory conformation, subsequent oligomerization of NOD1/2 and recruitment of the receptor-interacting protein kinases 2 (RIP2) via CARD–CARD interactions, and finally activation of the nuclear factor kappa-light-chain-enhancer of activated B-cells (NF-κB) and mitogen-activated protein kinases (MAPKs), which drive expression of pro-inflammatory genes and antimicrobial responses [13,14]. The NLRP subgroup comprises 14 members in humans, NLRP3 being one of the best-investigated molecules in this subgroup and involved in inflammasome activation. NLRP3 is activated by diverse stimuli including PAMPs, damage or danger-associated molecular patterns (DAMPs) and cellular perturbations such as endoplasmic reticulum (ER) and mitochondrial stress. Upon activation, NLRP3 recruits the adaptor apoptosis-associated speck-like protein containing a CARD (ASC) and the pro-Caspase-1, triggering Caspase-1 activation and subsequent cleavage of interleukin 1 beta (IL1β) and eventually cell death [15,16]. The central NOD domain has an ATPase activity and is essential for the oligomerization of NLRs. Despite conservation of their global functions and signaling pathways, specific NLRs show differences between mice and humans. For instance, NOD1/2 trigger autophagy in both human and mouse cells, yet their downstream adaptor RIP2 is required for autophagy induction only in human cells [17,18]. The CARD only protein (COP) and the inhibitory CARD (INCA) are negative regulators of IL1β production and these are present solely in the human genome [19,20]. Moreover, lipopolysaccharide triggers an alternative inflammasome involving the Toll-like receptor 4 (TLR4)-RIP1-Caspase-8 signaling upstream of NLRP3, which is unique to human monocytes [21]. These examples illustrate the necessity for a careful consideration of potential similarities and differences in NLR biology across species. Specific examples are highlighted in the context of cell stress in this review.

Sensing of PAMPs by PRRs initiates immune responses irrespective of microbial pathogenicity. Detection of patterns of pathogenicity or homeostasis-altering molecular processes (HAMP) enables fine-tuning of immune responses and allows for differentiation of pathogens from nonpathogenic microbes [22,23]. During millions of years of co-evolution, pathogens have developed mechanisms targeting host homeostatic functions, and the resulting perturbations permit pathogens to infect, replicate and spread to permissive hosts. Accumulating evidence indicates that disruption of cellular homeostasis elicits NLR activation. Thus, we propose that in addition to PAMP sensing, NLRs also detect stress-associated molecular patterns (SAMPs), allowing for the discrimination of pathogens from harmless microbes. We extend the SAMP concept from intercellular stress signals (e.g., alerting neighboring cells of oxidative stress [24]) to intracellular cues that signify pathogenicity and activate innate immunity. Accordingly, cellular stress as a trigger and regulator of host antimicrobial defense will be discussed in the context of infection with various pathogenic microbes ranging from viruses to protozoans.

## 2. Pathogen-Induced Perturbation of Cellular Homeostasis

Although different pathogens employ a broad pool of virulence factors to perturb cellular homeostasis, the outcome of host–pathogen interactions converges toward conserved overarching cellular processes, notably cytoskeleton disruption, ER stress, mitochondrial dysfunction and protein translation inhibition.

### 2.1. Disruption of Cytoskeleton Dynamics

**Figure 1:**
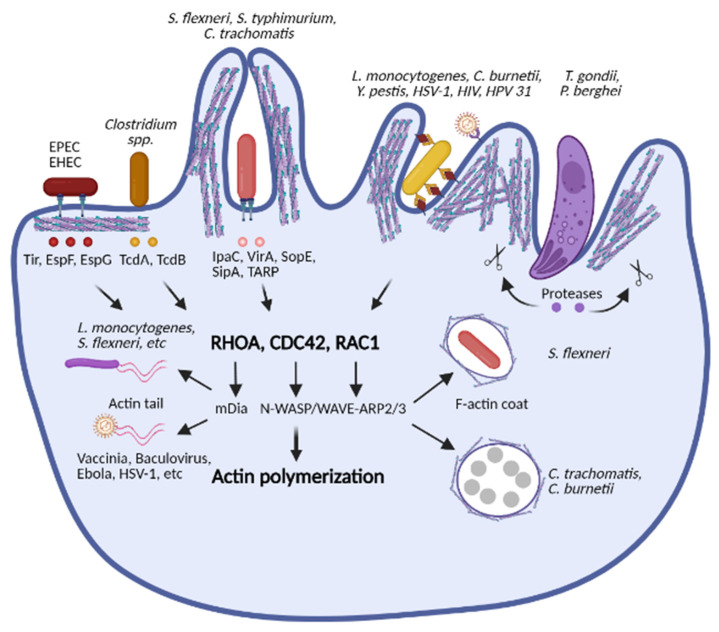
Pathogens cause cytoskeleton disruption. Bacteria and viruses subvert host cytoskeleton dynamics for their entry, intracellular survival and dissemination. They employ various effector molecules, toxins, or viral proteins to induce actin remodeling by manipulating Rho GTPases (RHOA, CDC42, and RAC1), which are the central regulators of the actin polymerization. Parasites such as *Toxoplasma gondii* and *Plasmodium berghei* also induce actin rearrangement by binding or cleaving actin-associated factors. Abbreviations: RHOA, Ras homolog family member A; CDC42, cell division control protein 42 homolog; RAC1, Ras-related C3 botulinum toxin substrate 1; mDia, mammalian homolog of Diaphanous; N-WASP, neuronal Wiskott-Aldrich Syndrome protein; WAVE, Wiskott-Aldrich syndrome protein family verprolin-homologous; ARP2/3, actin related protein 2/3; TirA, translocated intimin receptor A; EspF, enteropathogenic *E. coli* effector protein F; EspG, enteropathogenic *E. coli* effector protein G; TcdA, *Clostridium difficile* toxin A; TcdB, *C. difficile* toxin B; IpaC, invasion plasmid antigen C; VirA, virulence factor A; SopE, salmonella outer protein E; SipA, salmonella invasion protein A; TARP, type III secretion system actin-recruiting effector. *Image created with BioRender.com*.

In eukaryotic cells, the cytoskeleton network comprises three components: actin filaments, tubulin microtubules and intermediate filaments [25]. It plays critical roles in diverse cellular processes such as endocytosis and phagocytosis, intracellular transport, cell migration and division [25]. The cytoskeleton network organization, especially its assembly and disassembly, is tightly and precisely regulated. However, pathogens have developed diverse strategies to manipulate the cytoskeleton dynamics at different stages of infection (Figure 1).

Under physiological conditions, in response to extracellular stimuli, the Rho family of GTPases including RHOA, RAC1, and CDC42 is turned on and subsequently induces activation of the nucleating factor actin related protein 2/3 (ARP2/3), which drives actin polymerization [26,27]. These pathways are exploited by pathogens for actin-mediated adhesion and invasion. For instance, extracellular bacteria such as enterohemorrhagic *Escherichia coli* (EHEC) and enteropathogenic *E. coli* (EPEC) translocate various effectors into cells via a type III secretion system (T3SS) to trigger actin polymerization and pedestal formation for bacterial adhesion. The translocated intimin receptor (Tir) is critical for the adhesion of EHEC and EPEC by activating the ARP2/3 complex [28,29,30]. The enteropathogenic *E. coli* effector protein F (EspF) binds to actin, profilin, ARP2 and to neural Wiskott-Aldrich syndrome protein (N-WASP), which subsequently induces actin polymerization [31]. EspG binds to tubulin, causing microtubule depolymerization and actin stress fiber formation [32]. In contrast, *Clostridium difficile* utilizes TcdA and TcdB toxins to inactivate the Ras homolog family member A (RHO), the Ras-related C3 botulinum toxin substrate 1 (RAC1) and the cell division control protein 42 homolog (CDC42) via glycosylation, thereby causing actin depolymerization and gut permeability [33,34]. Intracellular pathogens require access to subcellular compartments in phagocytic or non-phagocytic cells to meet their metabolic requirements for replication. Accordingly, various bacterial pathogens have developed strategies for rapid entry by inducing cytoskeleton reorganization. For example, *Shigella flexneri* secretes the invasion plasmid antigen C (IpaC) via T3SS to induce formation of filopodial extensions, activation of CDC42 and RAC1 for lamellipodial extensions, and of the avian sarcoma (Schmidt-Ruppin A-2) viral oncogene homolog (Src) kinase for actin polymerization, thus facilitating bacterial internalization [35,36,37]. Furthermore, *Shigella* utilizes the virulence factor A (VirA) to inhibit tubulin polymerization and destabilize microtubules for its efficient entry into epithelial cells [38,39]. Similar to *Shigella*, *Salmonella typhimurium* secretes several effectors via T3SS to activate CDC42 and RAC1 [40,41,42,43]. Salmonella outer protein E (SopE) and SopE2 trigger membrane ruffling and actin rearrangement by stimulating GDP/GTP exchange activity of CDC42 and RAC1 [40,41]. Salmonella invasion protein A (SipA) directly binds to actin and enhances actin polymerization [43]. *Chlamydia trachomatis* also secretes the effector called translocated actin recruiting phosphoprotein (TARP) to induce actin polymerization during entry into non-phagocytic cells [44]. Other intracellular bacteria including *Listeria monocytogenes* [45], *Coxiella burnetii* [46] and *Yersinia pestis* [47] do not utilize protein-mediated membrane ruffling. Instead, they invade host cells via an active zippering mechanism in which actin rearrangement is also involved [48].

Many viruses exploit the host cytoskeleton for their entry, replication, cellular transport and egress. Here, we only focus on mechanisms by which viruses actively induce cytoskeleton remodeling. For example, Herpes Simplex Virus-1 (HSV-1) induces RHOA activation and subsequently promotes its entry via an unusual phagocytosis-like uptake [49]. The Env protein of Human Immunodeficiency Virus (HIV) induces RAC1 activation and further promotes ARP2/3-dependent actin polymerization for HIV fusion with the cell membrane [50]. The Sendai virus increases the level of the actin-modifying protein Villin, leading to actin polymerization and viral entry [51]. Human Papillomavirus type 31 (HPV 31) activates tyrosine and phosphoinositide 3-kinases (PI3K) to promote cytoskeletal rearrangement, which allows viral entry via filopodia transport [52].

For obligate intracellular parasites, plasma membrane and cytoskeleton form barriers against invasion, hence some parasites actively induce actin reorganization to facilitate their entry and release. *Toxoplasma gondii* and *Plasmodium berghei* trigger the formation of ring-shaped actin structures at the parasite–cell junction for host cell invasion [53]. *T. gondii* secretes the actin-binding protein toxofilin to regulate actin filament disassembly and turnover [54], whereas *P. falciparum* employs several proteases such as serine protease gp76 [55], chymotrypsin-like protease [56], plasmepsin II [57] and possibly the cysteine protease [58] to induce actin rearrangement by cleavage of various actin-associated factors [56,57,58].

After cell invasion, several pathogens hijack the cytoskeleton network to subvert intracellular killing mechanisms. *Shigella* induces the formation of a unique actin coat-like structure that inhibits bacterial killing by blocking fusion of the phagosomes with late endosomes/lysosomes [59,60]. This actin cocoon is triggered by the T3SS effector IcsB (Intra-inter-cellular spread B) by recruiting the actin nucleation machinery [60]. Massive cytoskeleton perturbation also occurs during intracellular replication of *C. trachomatis*. *Chlamydia* inclusions, which contain replicating bacteria, are enclosed in compact F-actin and intermediate filaments that cooperatively maintain integrity and stability of the inclusions [61]. *C. burnetii* replicates in large parasitophorous vacuoles that require F-actin association in a process depending on RHOA and CDC42 [62].

Several pathogens take advantage of actin-based motility for cell-to-cell spread. *L. monocytogenes* achieves such motility via the bacterial factor actin assembly-inducing protein (ActA) [63]. ActA mimics the ARP2/3 nucleation-promoting factor N-WASP. It binds to actin monomers and activates ARP2/3, thus promoting actin nucleation [64,65]. IcsA from *Shigella* recruits N-WASP and ARP2/3 on its surface to induce actin polymerization [66,67,68]. *Rickettsia parkeri* utilizes two actin-polymerizing proteins, RickA and surface cell antigen 2 (Sca2), for early and late motility, respectively [69]. Various *Burkholderia* species also show distinct actin-based motility mediated by Burkholderia intracellular motility A (BimA) orthologs by mimicking different host actin-polymerizing proteins [70]. The actin tails formed at the bacterial surface generate mechanical forces that propel the bacteria into adjacent cells via membrane protrusion [71]. Several viruses also manipulate the actin cytoskeleton to induce rapid cell-to-cell spread without free virion release. The Vaccinia virus employs the viral protein A36 [72,73] and the Baculovirus uses the P78/83 capsid protein [74] to promote ARP2/3-dependent actin polymerization. Actin tails are also found to be associated with Ebola virus nucleocapsids, contributing to its budding [75]. Pseudorabies virus (PRV) and HSV-1 induce cytoskeletal rearrangements and cell extensions to facilitate viral spread by US3 kinase [76,77]. The P protein of Human Metapneumovirus (HMPV) promotes CDC42, RAC1 and RHOA dependent formation of intercellular actin extensions, thus contributing to direct cell-to-cell spread [78].

In sum, bacterial, viral and protozoal pathogens employ unrelated virulence factors that target actin, tubulin and intermediate filaments to induce host cytoskeleton remodeling for their invasion, intracellular survival and exit from host cells. The dysregulated cytoskeleton dynamics results in the perturbation of homeostatic functions and signifies pathogenicity during infection.

### 2.2. ER Stress

The ER maintains cellular homeostasis by regulating protein folding and processing, lipid synthesis and calcium storage and release. Accumulation of misfolded proteins in the ER lumen leads to its functional impairment and ER stress [79,80]. To counteract the detrimental effects of the ER stress, eukaryotic cells initiate the unfolded protein response (UPR) pathways. The ER stress is sensed by three ER transmembrane receptors: protein kinase R-like ER kinase (PERK), inositol-requiring enzyme 1α (IRE1α) and activating transcription factor 6 (ATF6). Under homeostatic conditions, these sensors are inactive due to interactions with the ER luminal heat shock protein 70 (HSP70)-type chaperone binding immunoglobulin protein (BiP). Upon ER stress, BiP dissociates from these sensors and binds to misfolded proteins in the ER, thereby releasing the sensors to initiate the three branches of UPR [79,80].

**Figure 2:**
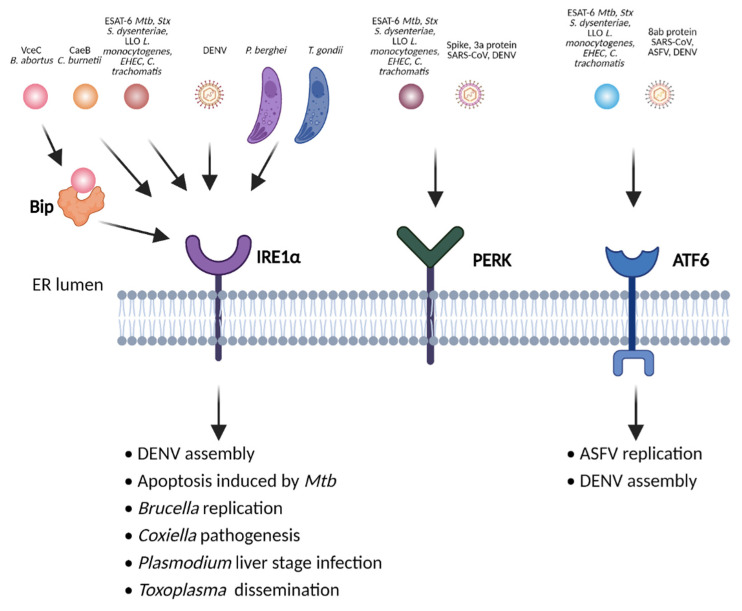
Various pathogens cause ER stress to modulate host cell death or facilitate their replication and dissemination. Unfolded protein responses (UPR) are mediated by the ER stress sensors protein kinase R-like ER kinase (PERK), inositol-requiring enzyme 1α (IRE1α) and activating transcription factor 6 (ATF6). The 6 kDa early secretory antigenic target (ESAT-6) from *M. tuberculosis* (Mtb), shiga toxin 1 (Stx) from *S. dysenteriae*, listeriolysin O (LLO) from *L. monocytogenes*, dengue virus (DENV), etc. induce all three branches of UPR. VceC, a T4SS effector from *B. abortus*, binds to the ER chaperon binding immunoglobulin protein (BiP) and triggers IRE1α signaling. CaeB (*C. burnetii* anti-apoptotic effector B) from *C. burnetii* stimulates IRE1α signaling, facilitating its pathogenesis. The severe acute respiratory syndrome coronavirus (SARS-CoV) induces PERK and ATF6 signaling and the African Swine Fever Virus (ASFV) activates only ATF6. *Image created with BioRender.com*.

Diverse pathogens disrupt ER homeostasis and cause ER stress, thus facilitating their survival and replication and controlling host cell death (Figure 2). Viruses exploit the ER for their protein synthesis and processing. Hence, a large number of viruses can induce ER stress to promote their replication, twist host cell death and facilitate their dissemination [81]. For instance, the African Swine Fever Virus (ASFV) utilizes ER as replication sites. It activates ATF6-dependent UPR to promote viral replication [82,83]. The Severe Acute Respiratory Syndrome Coronavirus (SARS-CoV) induces activation of PERK via its spike [84] and 3a protein [85] as well as the activation of ATF6 via the 8ab protein [86]. Dengue Virus (DENV) triggers IRE1α, PERK and ATF6-mediated UPR to promote virion assembly and alleviate virus-induced apoptosis [87,88,89,90].

Various bacteria and parasites also trigger ER stress, contributing to the pathogenesis of infection. The 6 kDa early secretory antigenic target (ESAT-6), a secreted virulence factor from *Mycobacterium tuberculosis* (*Mtb*), activates IRE1α and PERK mediated UPR, resulting in Ca^2+^ release, reactive oxygen species (ROS) production, and subsequent apoptosis [91]. Heparin-binding hemagglutinin antigen (HBHA) from *Mtb* also induces ER stress through cytosolic Ca^2+^ and ROS generation, leading to apoptosis [92]. Shiga toxin 1 from *S. dysenteriae*, the cholesterol-dependent cytolysin Listeriolysin O (LLO) from *L. monocytogenes* as well as EHEC and *C. trachomatis* activate all ER stress sensors IRE1α, PERK, and ATF6 [93,94,95,96]. *B. abortus* resides in ER-derived vacuoles and VceC, an effector of T4SS, directly interacts with the ER chaperon BiP and activates the IRE1α- X-box binding protein 1 (XBP1) pathway [97]. The cyclic dinucleotide c-di-GMP from *B. abortus* also triggers stimulator of interferon genes (STING)-dependent ER stress responses, which facilitate bacterial replication in vivo [98]. Streptolysins O and S from group A *Streptococcus* (GAS) elicit ER stress, and promote biofilm formation and dissemination within soft tissues in vivo [99]. *C. burnetii* stimulates IRE1α signaling and inhibits ER stress-induced apoptosis via the T4SS effector CaeB (*C. burnetii* anti-apoptotic effector B), likely contributing to pathogenicity in vivo [100]. *P. berghei* induces IRE1α-mediated UPR, promoting liver stage infection [101], while *T. gondii* activates the same ER stress sensor to enhance its dissemination in vivo [102]. Altogether, viral, bacterial or parasitic pathogens often induce ER stress to promote their replication and dissemination. Their abilities to induce ER stress distinguish them from non-pathogenic microbes.

### 2.3. Mitochondrial Dysfunction

**Figure 3:**
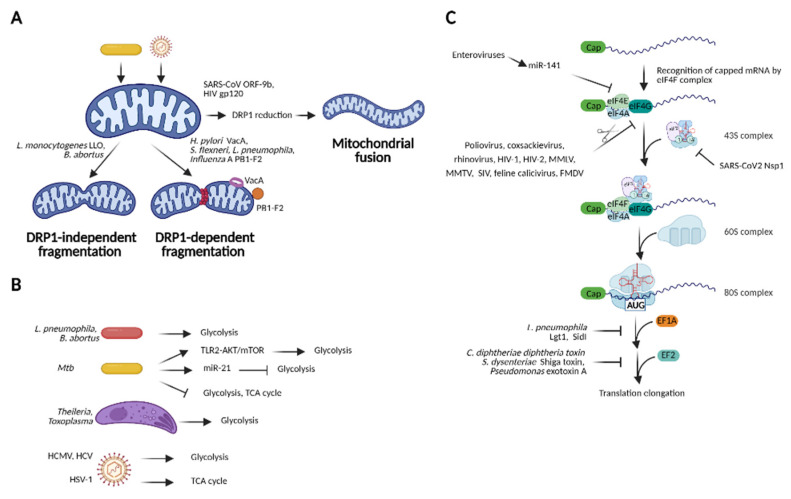
Mitochondrial dysfunction and blockade of host translation induced by pathogens. (**A**) Manipulation of mitochondrial dynamics by pathogens. Pathogenic viruses and bacteria cause mitochondrial fragmentation or mitochondrial fusion for replication, persistence in host cell or dissemination; (**B**) Modulation of host metabolism by pathogens. To acquire the nutrient from the host, pathogens trigger glycolysis or modulate the TCA cycle dependent on their metabolic requirements; (**C**) Inhibition of host translation by pathogens. To shut off host defense, many viruses such as poliovirus, coxsackievirus and rhinovirus, HIV-1, enteroviruses, and SARS-CoV2 target the eIF4F complex to inhibit translation initiation, while some bacterial pathogens utilize effector proteins or toxins to inactivate translation elongation factors-EF1A and EF2. Abbreviations: DRP1, dynamin related protein 1; VacA, vacuolating cytotoxin A; LLO, listeriolysin O; TLR2, toll-like receptor 2; AKT, protein kinase B; mTOR, mechanistic target of Rapamycin; TCA cycle, tricarboxylic acid cycle; HCMV, Human Cytomegalovirus; HCV, Hepatitis C Virus; HSV-1, Herpes simplex virus 1; HIV, Human immunodeficiency virus; MMLV, Moloney murine leukemia virus; MMTV, Mouse mammary tumor virus; SIV, Simian immunodeficiency virus; FMDV, Foot-and-mouth disease virus; eIF, eukaryotic translation initiation factor; EF1A/1B, eukaryotic translation elongation factor 1A/1B; Lgt1, Legionella glucosyltransferase 1; SidI, substrate I of Icm/Dot transporter; Nsp1, nonstructural protein 1. *Image created with BioRender.com*.

Mitochondria are central organelles for many homeostatic processes including oxidative phosphorylation and ATP production, fatty acid oxidation and cell death. They are also crucial for innate immunity and inflammatory responses [103,104]. RNA sensors (e.g., retinoic acid-inducible gene I (RIG-I) and melanoma differentiation-associated protein 5 (MDA5)) converge their signals toward mitochondrial antiviral-signaling protein (MAVS), which triggers TANK-binding kinase 1 (TBK1)/Interferon regulatory factor 3 (IRF3)-dependent type I interferon (IFN-I) signaling [105,106,107,108]. Mitochondria-derived ROS also facilitate bacterial killing [109,110]. Moreover, metabolic reprogramming of immune cells in mitochondria governs immune responses and inflammation [111,112,113,114]. Therefore, it is not surprising that many pathogens have developed strategies to provoke mitochondrial dysfunction (Figure 3) and thus escape the host defense.

Mitochondria are dynamic organelles that constantly undergo fission and fusion and their dynamics are important for processes such as energy production, mitochondrial quality control and cell death. Manipulation of mitochondrial dynamics, either fragmentation or elongation, has been reported as pathogenicity mechanism for bacterial and viral pathogens (Figure 3A). *L. monocytogenes* elicits mitochondrial fragmentation via multiple mechanisms. The pore-forming toxin LLO causes loss of mitochondrial membrane potential and reduces ATP generation [115], increases expression of MIC10, a critical component of the mitochondrial contact site and cristae organizing system (MICOS) complex [116], and induces mitochondrial fission independent of the dynamin related protein 1 (DRP1) [117]. Altering dynamics of mitochondrial fusion during infection affects survival of *Listeria*, suggesting that it manipulates mitochondria dynamics to establish an infection [115]. *B. abortus* induces a drastic mitochondrial fragmentation independent of DRP1 [118], whereas *H. pylori* utilizes the vacuolating cytotoxin A (VacA) to elicit mitochondrial fragmentation via a mechanism dependent on DRP1 [119]. Similarly, *S. flexneri* and *L. pneumophila* induce DRP1-dependent mitochondrial fragmentation for their intracellular replication [120,121], whereas Influenza A Virus (IAV) employs the viral protein PB1-F2 to cause such fragmentation. Moreover, PB1-F2 is imported to mitochondria via the translocase of the outer membrane 4 (TOM4) and inhibits RIG-I/MVAS-mediated IFN-I response [122]. Other viral pathogens rather stimulate mitochondrial fusion. For instance, the open reading frame-9b (ORF-9b) from SARS-CoV triggers ubiquitination and proteasomal degradation of DRP1, resulting in mitochondrial elongation, degradation of MAVS, TNF receptor-associated factor (TRAF3) and TRAF6 and inhibition of IFN-I [123]. The glycoprotein 120 (gp120) of HIV induces mitochondrial fusion by reducing total and active DRP1 levels [124].

Various pathogens are also reported to modulate the host metabolism to ensure a replication niche (Figure 3B). *Mtb* induces upregulation of key glycolytic enzymes while lowering expression of enzymes involved in the tricarboxylic acid (TCA) cycle and oxidative phosphorylation in murine lungs, suggesting a metabolic shift from oxidative phosphorylation to aerobic glycolysis in tuberculosis [125]. *Mtb* also increases glycolysis in human and mouse macrophages in vitro, in turn contributing to bacterial control via IL1β release [126]. This metabolic shift toward glycolysis is initiated by the toll-like receptor 2 (TLR2) activation and mediated by the protein kinase B/mechanistic target of the rapamycin (AKT/mTOR) pathway [127]. *Mtb* augments expression of microRNA-21 (miR-21) in macrophages, which in turn dampens glycolysis by targeting phospho-fructo-kinase, muscle isoform (PFK-m). IFNγ enhances PFK-m expression, thereby contributing to improved control of *Mtb* [128]. Opposingly, *Mtb* promotes a quiescent energy phenotype with reduced glycolysis and TCA cycle in human monocyte-derived macrophages, while attenuated *M. bovis* BCG or dead *Mtb* induce glycolysis [129]. These apparently contradictory findings may be attributed to host-, cell type-, or infection stage-specific metabolic remodeling during *Mtb* infection. Other intracellular bacteria (e.g., *L. pneumophila* and *B. abortus*) trigger a metabolic switch toward glycolysis to support bacterial survival [121,130]. During *S. flexneri* infection, host cells convert glucose into acetate instead of lactate, and the bacteria capture the downstream metabolite pyruvate for their growth [131]. Similarly, the Apicomplexa parasite *Theileria* induces aerobic glycolysis in bovine leukocytes [132,133] by stabilizing host pyruvate kinase isoform M2 (PKM2) [134]. To utilize host glucose as a carbon source, *T. gondii* also promotes glycolysis in murine dendritic cells [135] and in fibroblasts [136,137] by increasing the expression of enzymes involved in glycolysis [135,136,137]. *T. cruzi* enhances host glucose uptake to fuel its metabolism, yet without affecting glycolysis [138]. Instead, the protozoa *P. falciparum* hampers glycolysis in red blood cells due to oxidative stress [139]. Viruses completely rely on host metabolism to fuel their replication, however, some of them are able to actively subvert host metabolism for their benefit. The infection with the Human Cytomegalovirus (HCMV) increases the glycolytic flux, while HSV-1 induces the TCA cycle for its replication [140]. The Hepatitis C Virus (HCV) infection increases glycolysis and activates the pentose phosphate pathway as well as lipid synthesis for virus replication at the early stage, and fatty acid oxidation at later stages [141].

Overall, these findings strengthen the concept that mitochondrial dysfunction represents an evasion mechanism that facilitates efficient escape from host defense during viral, bacterial and protozoal infection.

### 2.4. Blockade of Protein Translation

Viral replication completely depends on the host’s translation machinery. To facilitate translation of viral proteins and restrict production of host antiviral proteins, viruses have developed multiple strategies to globally block host translation (Figure 3C). Translation of host mRNA occurs in a cap-dependent manner and the eukaryotic initiation factor 4F (eIF4F) complex consisting of the cap-binding protein eIF4E, the scaffold eIF4G, and the RNA helicase eIF4A is critical for efficient targeting of ribosomes to the m^7^G cap [142,143]. Many viruses directly target the eIF4F complex given the importance of the complex in host mRNA translation [144]. Poliovirus [145], coxsackievirus and rhinovirus [146], HIV-1 [147], HIV-2, moloney murine leukemia virus, mouse mammary tumor virus and simian immunodeficiency virus [148], feline calicivirus [149] and foot-and-mouth disease virus [150] encode various proteases to directly degrade eIF4G. The rotavirus non-structural protein 3 (NSP3), a homolog of the poly(A) binding protein (PABP), interacts with eIF4G and disrupts interaction between eIF4G and PABP, thus shutting off host protein synthesis while keeping viral protein translation unaltered [151]. Enteroviruses induce expression of miR-141 that targets eIF4E mRNA for its degradation [152]. NSP1 from SARS-CoV-2 binds to the 40S ribosomal subunit, leading to the blockade of mRNA entry and disruption of host translation [153].

Although bacteria do not depend on the host translation machinery, some species also develop strategies to impair host translation and inhibit the immune defense (Figure 3C). Exotoxins (e.g., diphtheria toxin from *C. diphtheriae*, shiga toxin from *S. dysenteriae* and exotoxin A from *P. aeruginosa*) block protein translation by inactivating the host elongation factor 2 (EF2) via ADP-ribosylation [154]. *C. trachomatis* substantially inhibits host protein synthesis, possibly by inducing ER or mitochondrial stress [155]. *L. pneumophila* uses several effectors to interfere with host protein translation. Legionella glucosyltransferase 1 (Lgt1) inactivates the elongation factor 1-alpha (EF1A) [156], the substrate I of Icm/Dot transporter (SidI) binds to and disables EF1A and EF1Bγ [157], and Legionella kinase 4 (LegK4) phosphorylates all members of the HSP70 family (HSP70, HSP72, and BiP) and compromises folding of nascent polypeptides [158,159]. Collectively, although host translation is primarily targeted by viruses, selected bacterial pathogens employ multiple toxins or effectors to inhibit protein translation.

## 3. NLRs Activation by Perturbation of the Cellular Homeostasis

Several NLR members can be activated by PAMPs, DAMPs and also by pathogen-induced perturbation of cellular homeostasis, possibly via SAMP generation. Members of two NLR subclasses, NLRC and NLRP, are major receptors involved in inflammation induced by disrupted cellular homeostasis during pathogen invasion. Involvement of NOD1 and NOD2 as well as of NLRP1 and NLRP3 in sensing cellular homeostasis dysfunction has been recently demonstrated [160,161,162,163]. These NLR members are endowed with remarkable multitasking abilities of directly sensing microbial moieties, detecting danger cues and monitoring cellular stress during infection.

### 3.1. NOD1 and NOD2 Detect the Disruption of Cytoskeleton Dynamics

**Figure 4:**
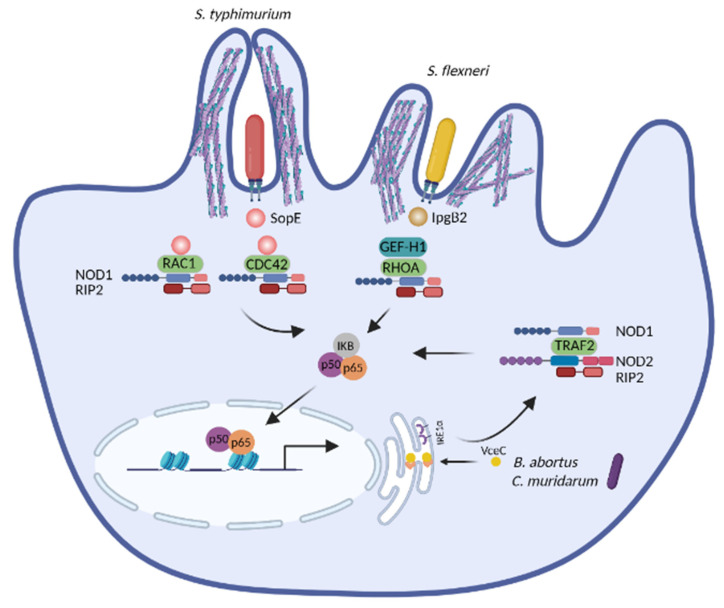
NOD1 and NOD2 trigger NF-κB activation by detecting pathogen-induced cytoskeleton disruption or pathogen-induced ER stress. Virulence factors from *S. typhimurium* and *S. flexneri* induce aberrant actin polymerization, subsequently triggering NOD1-mediated NF-κB activation. *B. abortus* and *C. muridarum* elicit ER stress, which is further sensed by NOD1 and NOD2 to enable NF-κB activation. Abbreviations: RHOA, Ras homolog family member A; CDC42, cell division control protein 42 homolog; RAC1, Ras-related C3 botulinum toxin substrate 1; GEF-H1, guanine nucleotide exchange factor H1; TRAF2, TNF receptor associated factor 2; RIP2, receptor-interacting-serine/threonine-protein kinase 2; IKB, inhibitor of κB; IRE1α, inositol-requiring enzyme-1a; IpgB2, invasion plasmid gene B2; SopE, *Salmonella* outer protein E; VceC, virB-coregulated effector C. *Image created with BioRender.com*.

Several studies have unveiled that NOD1 and NOD2 are activated upon cytoskeleton disruption [164,165,166,167]. Experiments with chemical inhibitors have first indicated that actin-depolymerizing agents (e.g., cytochalasin D and latrunculin B) and actin-polymerizing compounds (e.g., jasplakinolide) induce NF-κB activation and IL8 production in a process depending on NOD2 [164,165,168]. RAC1 interacts with NOD2 and expression of a RAC1 dominant negative mutant leads to loss of membrane ruffles and dissociation of NOD2 from RAC1, further enhancing NOD2-mediated NF-κB activation [164]. Consistently, expression of the dominant negative RAC1 mutant triggers NOD2/RIP2-dependent NF-κB activation [169]. Many pathogens manipulate RHO GTPases to induce cytoskeleton rearrangement and host invasion [36,38,40,41,42] and these processes promote NOD1 and/or NOD2 mediated immune activation (Figure 4). The T3SS effector SipA from *Salmonella* requires NOD1 and NOD2 as well as their downstream adaptor RIP2 to trigger NF-κB activation. The actin-binding domain of SipA is dispensable for NF-κB activation, suggesting that its ability to cause actin rearrangement is obsolete for the NF-κB effects [166]. SipA also induces redistribution of LAMP1-positive compartments toward the microtubule-organizing center (MTOC) [170]. Thus, SipA-induced NOD1 and NOD2 activation is likely connected with its ability to manipulate the microtubule network. Another T3SS effector, SopE from *Salmonella*, a nucleotide-exchange factor, causes membrane ruffling by activating RHO GTPases [40]. It further promotes the interaction of NOD1 with RAC1 and CDC42, which in turn triggers NOD1/RIP2-mediated NF-κB activation. In this case, NOD1 likely senses bacteria-induced aberrant activation of RHO GTPases [167]. Another cytoskeleton-related molecule activating NOD1 is the guanine nucleotide exchange factor H1 (GEF-H1). GEF-H1 stimulates the formation of actin stress fibers by activating RHOA upon its dissociation from microtubules [171] and is required for NOD1 canonical ligand-induced NF-κB activation, however, independent of RHOA [172]. GEF-H1 also contributes to NOD2-induced NF-κB activation and cytokine production by mediating the phosphorylation of RIP2 [173]. In the context of *S. flexneri* infection, IpgB2, a T3SS effector, binds to the mammalian homolog of Diaphanous (mDia) and directly induces actin polymerization by functioning as an analog of active RHOA [35]. In line with its role in actin polymerization, IpgB2 localizes to actin-associated cellular junctions when expressed in cells. Ectopic expression of the invasion plasmid gene B2 (IpgB2) or outer *Shigella* protein B (OspB), another T3SS effector without a known role in cytoskeleton remodeling, induces GEF-H1 and NOD1-mediated NF-κB activation, yet is independent of the classical NOD1/2 downstream signaling component RIP2 [172]. Other molecules interfering with NOD1 activation are Cofilin and Cofilin phosphatase slingshot homolog 1 (SSH1), which control the disassembly of actin filaments. Both are essential for maintaining actin cytoskeleton dynamics and inducing NF-κB activation upon stimulation with *Shigella* and NOD1 agonists [174]. As RHOA negatively regulates Cofilin, it seems unlikely that NOD1 activation is connected with excessive RHO activation under these conditions.

Altogether, pathogens exploit various mechanisms to hijack the cytoskeleton dynamics. During infection, NOD1 and/or NOD2 represent an intracellular surveillance system inducing NF-κB activation by detecting cytoskeleton disruption, instead of monitoring RHO GTPases. The detailed mechanisms by which NOD1 and NOD2 monitor cytoskeleton dynamics and induce NF-κB activation need further elucidation.

### 3.2. NOD1 and NOD2 Sense the ER Stress during Infection

Chemical agents that cause ER stress such as tunicamycin, brefeldin A, 2-deoxyglucose and thapsigargin (TG) trigger an NF-κB-dependent inflammation. However, the ER stress induced UPR is uncoupled from NF-κB activation, suggesting that ER stress activates two distinct signaling pathways: the classical UPR and the NF-κB activation [175]. Further investigations have linked ER stress-induced NF-κB activation with the intracellular PRRs NOD1/2 [160]. IL6 production upon TG stimulation is impaired by NOD1/2 knockout (KO), demonstrating NOD1/2 activation by ER stress. The ER stress inhibitor tauroursodeoxycholic acid (TUDCA) and the IRE1α inhibitor KIRA6 (IRE1α kinase inhibiting RNase attenuator 6) block TG-induced IL6 production in vitro and *in vivo*, while both inhibitors have no impact on IL6 production stimulated by MDP, the canonical bacterial agonist of NOD2. Thus, the ER stress and bacterial PAMPs employ different signaling pathways to activate NOD1/2 [160]. In the context of infection, the *B. abortus* T4SS effector virB-coregulated effector C (VceC) induces ER stress through interaction with the chaperon BiP [97] and triggers NOD1/2- and RIP2-dependent IL6 production. Infection of NOD1/2 KO mice with wildtype of *B. abortus* or infection of wildtype mice with the VceC null mutant yields decreased IL6 production, milder pathology and increased survival of mice. Therefore, the ER stress elicited by *B. abortus* infection activates NOD1/2-dependent inflammation, hence contributing to the pathogenesis of brucellosis [160]. *C. muridarum* infection also induces ER stress and NOD1/2-RIP2-mediated IL6 production [160], however, inhibition of ER stress or depletion of NOD1/2 or RIP2 leads to increased bacterial burdens *in vivo*. Thus, the ER stress and subsequent NOD1/2-dependent inflammation caused by *Chlamydia* opposingly contributes to infection clearance [96]. Furthermore, the ER stress induced by TG significantly increases NF-κB activation and the expression of IL6 and IL23 in response to NOD1 stimulation upon *S. enterica* infection in vitro. However, the impact of the ER stress during *S. enterica* infection in vivo remains to be elucidated [176]. Together, NOD1 and NOD2 activate inflammatory responses by detecting the ER stress caused by various pathogens (Figure 4), yet the outcome of ER stress-induced NOD1/2 activation is disease-dependent. Given that many pathogens induce ER stress [81] and that peptidoglycan-free viruses and parasites activate NOD1 and/or NOD2-dependent inflammation [177], it is reasonable to speculate that NOD1 and/or NOD2 are activated by sensing the ER stress and possibly SAMPs during these infections. Additionally, aberrant ER stress is associated with many noncommunicable diseases such as neurodegenerative disorders, atherosclerosis, type 2 diabetes, and cancers [178]. Hence, it is worthwhile to further investigate whether the ER stress-induced inflammation in patients suffering from these diseases confers susceptibility to specific infections.

The molecular events that integrate ER stress with NOD1/2 signaling and NF-κB activation are a focus of current research. Upon ER stress, IRE1α binds to TRAF2, induces its oligomerization and the activation of NF-κB and c-Jun N-terminal kinases (JNK) [160,179]. Although NOD1 and NOD2 contain motifs predicted to bind TRAF2, evidence for a direct interaction of TRAF2 with NOD1/2 is missing. Moreover, whether kinase activity of IRE1α or other factors alter the putative interaction of TRAF2 and NOD1/2 awaits to be elucidated. A study demonstrates that ER stress triggered by TG results in Ca^2+^ efflux and subsequent Ca^2+^-dependent NOD1/2-mediated inflammation. Peptidoglycan contaminants in the serum could be internalized via endocytosis upon ER stress and subsequently activate NOD1 and NOD2 [180]. However, whether such trace amounts of peptidoglycan, or possibly other ligands, in the serum activate NOD1/2 requires further investigations. Recently, we have uncovered that generation of the endogenous metabolite sphingosine-1-phosphate (S1P) is remarkably increased upon various types of stress including ER stress and cytoskeleton disruption elicited by chemicals. However, other metabolites in the sphingolipid pathway (i.e., Ceramide and Sphinganine) are not affected by the same stimulations. S1P, but not Ceramide, directly binds to NOD1 and NOD2, and S1P delivery into cells leads to NOD1 or NOD2 mediated NF-κB activation. Hence, we propose that NOD1/2 detect perturbation of cellular homeostasis through sensing of the cytosolic metabolite S1P, which represents a SAMP [181]. Whether the S1P-NOD1/2 axis is activated during infection with peptidoglycan-free pathogens needs to be elucidated.

### 3.3. Activation of the NLRP3 Inflammasome by the ER Stress

**Figure 5:**
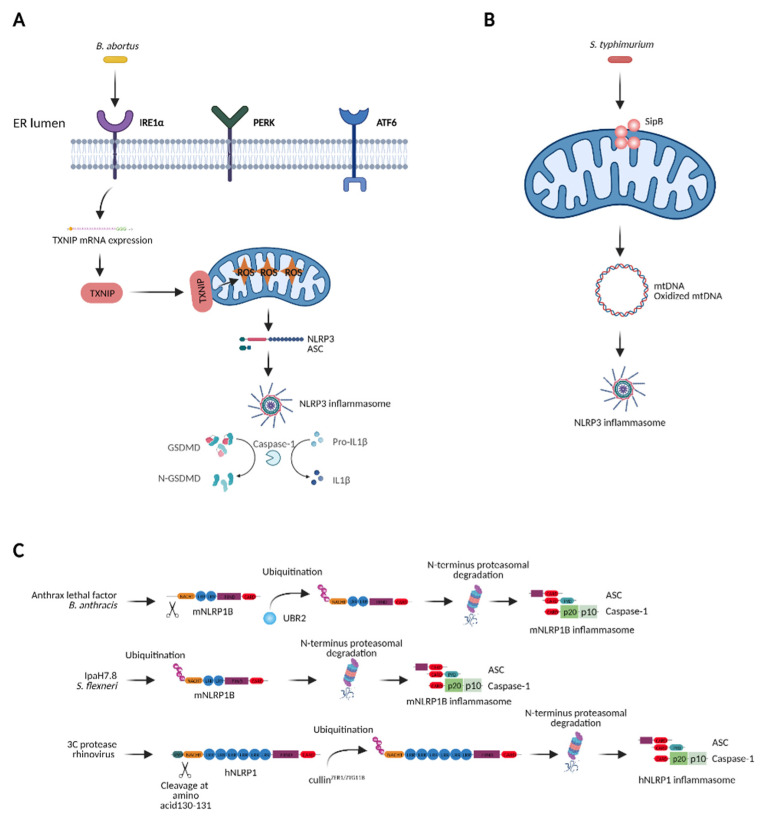
Distinct NLRP inflammasomes are activated by ER stress, mitochondrial dysfunction or directly by pathogens. (**A**) *B. abortus* induces ER stress, which causes NLRP3 inflammasome activation. (**B**) *S. typhimurium* targets mitochondria to trigger NLRP3 inflammasome activation. (**C**) Murine and human NLRP1 (mNLRP1B, hNLRP1) are targeted by pathogen virulence factors. Abbreviations: PERK, PKR-like ER kinase; IRE1α, inositol-requiring enzyme 1α; ATF6, activating transcription factor 6; TXNIP, thioredoxin-interacting protein; ASC, apoptosis-associated Speck-like protein containing a CARD; GSDMD, Gasdermin D; UBR2, ubiquitin protein ligase E3 component N-Recognin 2; ROS, reactive oxygen species; mtDNA, mitochondrial DNA; SipB, *Salmonella* invasion protein B; IpaH7.8, invasion plasmid antigen H7.8. *Image created with BioRender.com*.

Multiple studies have revealed that the ER stress triggered by chemical agents induces NLRP3 inflammasome activation, however, NLRP3 activation elicited by pathogen-induced ER stress has rarely been reported. Chemically induced ER stress (i.e., by tunicamycin or brefeldin A) causes activation of the NLRP3 inflammasome. This effect is uncoupled from the stress sensors IRE1α, PERK and ATF6α, but is dependent on ROS and potassium efflux that are mediated by the voltage-dependent anion-selective channel 1 (VDAC1), which transports metabolites and ions into mitochondria. Thus, ER stress-induced inflammasome activation and UPR are divergent processes under these circumstances [182]. Mechanistically, the ER stress induces the expression of thioredoxin-interacting protein (TXNIP) via PERK and IRE1α-mediated signaling pathways. TXNIP induces IL1β mRNA transcription and further activates the NLRP3 inflammasome for IL1β release [183]. Moreover, the ER stress also increases TXNIP mRNA by reducing the levels of the TXNIP destabilizing micro-RNA miR-17 via IRE1α [184] and by ROS formation [185], further leading to NLRP3 inflammasome activation. Thus, the IRE1α-TXNIP axis mediates activation of the NLRP3 inflammasome-subsequent ER stress, and might serve as a therapeutic target for metabolic disorders [186,187]. In the context of infection, *B. abortus* induces ER stress and subsequently NLRP3 inflammasome activation. In this circumstance, TXNIP expression is elevated and it translocates to mitochondria, resulting in increased mitochondrial ROS and mitochondrial damage, which result in NLRP3 inflammasome activation [188] (Figure 5A). Collectively, the chemical- or pathogen-induced ER stress activates the NLRP3 inflammasome via TXNIP, a process involving mitochondrial alterations. Whether other pathogens cause ER stress and this type of cell stress engages similar molecular pathways for IL1β production remains to be investigated.

### 3.4. NLRP3 Inflammasome Induced by Mitochondrial Dysfunction

Mitochondrial dysfunction leads to activation of the NLRP3 inflammasome [189,190] (Figure 5B). Inhibition of complex I, one of the key enzymes of the respiratory chain, results in robust production of mitochondrial ROS causing NLRP3 inflammasome activation [163]. VDAC1/2, which is critical for mitochondrial ROS generation, is required for the inflammasome induction by inhibiting complex I [163]. Consistently, stimulation with the TLR7 ligands imiquimod and CL097 results in ROS production and activation of the NLRP3 inflammasome [191]. Mitophagy is essential for maintaining mitochondrial homeostasis [192]. Defects in autophagy induce accumulation of damaged mitochondria and elevated NLRP3 inflammasome activation that is dependent on both mitochondrial ROS and release of mitochondrial DNA (mtDNA) in the cytosol [190,193,194]. Mitochondrial dysfunction also induces release of oxidized mtDNA, which directly binds to and activates the NLRP3 inflammasome [195]. The replication of mtDNA is dependent on the cytidine/uridine monophosphate kinase 2 (CMPK2), which further contributes to the production of oxidized mtDNA and subsequent NLRP3 activation [196]. In the context of infection, the T3SS effector *Salmonella* invasion protein B (SipB) from *S. typhimurium*, localizes to mitochondria causing their swelling and depolarization, which subsequently induces oxidized mtDNA-mediated NLRP3 inflammasome [195,197]. Some antibiotics also disturb mitochondrial functions due to the resemblance between mitochondria and bacteria. Linezolid, an oxazolidinone antibiotic, induces NLRP3 inflammasome activation by promoting mitochondrial dysfunction. The mitochondrial lipid cardiolipin recruits NLRP3 to the mitochondria by direct binding and stimulates NLRP3 activation [198]. Collectively, mitochondrial dysfunction triggers NLRP3 inflammasome activation via mitochondrial ROS, mtDNA, oxidized mtDNA or cardiolipin. Whether such pathways generally participate in pathogen-induced NLRP3 inflammasome activation requires further elucidation.

### 3.5. NLRP1 Inflammasome and Cellular Homeostasis

The mouse NLRP1B (mNLRP1B) was first identified as a critical component conferring susceptibility to the anthrax lethal factor (LF). LF-induced cell death is mediated by Caspase-1, suggesting that mNLRP1B mediates pyroptosis by LF [199]. The N-terminal proteolysis is crucial for LF-induced mNLRP1B inflammasome activation [200]. Similarly, the 3C protease of human rhinovirus directly cleaves and activates human NLRP1 (hNLRP1) inflammasome [162]. After cleavage by LF, mNLRP1B generates a new N-terminus recognized by UBR2, leading to the ubiquitination of mNLRP1B and degradation by the N-end rule pathway, which are critical for mNLRP1B inflammasome activation [161,201]. The *Shigella* effector invasion plasmid antigen H7.8 (IpaH7.8), an E3 ubiquitin ligase, also activates mNLRP1B inflammasome by directly ubiquitinating mNLRP1B for degradation [161]. In the case of hNLRP1, the glycine-specific N-degron machinery cullin^ZER1/ZYG11B^ mediates recognition and degradation of hNLRP1 subsequent cleavage by the 3C protease of human rhinovirus [162]. Therefore, functional degradation of NLRP1 allows the host immune system to distinguish pathogens by monitoring the downstream cellular damage [202]. In addition to pathogen-induced activation, Val-boroPro (VbP), an inhibitor of dipeptidyl peptidases 8 and 9 (DPP8/9), also triggers both mNLRP1B and hNLRP1 inflammasome activation [203]. Both the peptidase activity of DPP9 and its binding to hNLRP1 are required for hNLRP1 activation by VbP [204]. Therefore, it has been proposed that hNLRP1 may indirectly sense the perturbation of cellular homeostasis induced by DPP8/9 cleavage [205]. In line with this, 2-deoxyglucose (2DG), a glycolysis inhibitor, and sodium azide, an inhibitor of the mitochondrial electron transport chain, activate the mNLRP1B inflammasome, suggesting a link between ATP production and mNLRP1B activation. Indeed, ATP depletion by glucose starvation or hypoxia induces the activation of the mNLRP1B inflammasome [206]. Altogether, NLRP1 may sense the disruption of cellular homeostasis induced by DPP8/9 cleavage, cellular ATP depletion, or infections (Figure 5C). Elucidation of the substrate of DPP8/9 will shed light on the mechanism of NLRP1 activation by disruptions of cellular homeostasis.

## 4. Conclusions and Perspectives

Solely detecting PAMPs or DAMPs appears insufficient for accurate identification of pathogens by the immune system. First, PAMPs are present in commensal, pathobionts, and pathogenic microorganisms. Second, DAMPs can be produced upon pathogen-induced damage or during sterile tissue damage. DAMPs such as ATP, F-actin and DNA binding proteins (e.g., High-mobility group box 1 (HMGB1)) are host molecules released from damaged cells [207]. Hence, the immune system requires additional mechanisms to specifically detect the different types of damage and accordingly fine-tune the antimicrobial responses. Pathogens subvert host defense mechanisms by manipulating various cellular pathways, which in turn often leads to perturbation of cellular homeostasis. In plants, NLRs recognize directly or indirectly pathogen effector proteins or abiotic stress, resulting in effector-triggered immunity [208]. In line with this, detection of perturbations of cellular homeostasis by NLRs may also represent an ancient mechanism of sensing infection in animals. Detection of perturbations of cellular homeostasis enables the immune system to distinguish pathogens from nonpathogenic microbes and adjust the magnitude of immune response. Depending on the extent and types of stress, the innate immune system could activate different pathways, for instance, NOD1/2-mediated NF-κB activation and NLRPs-mediated pyroptosis. Thus, according to the severity of the damage, the innate immune system could adapt and apply appropriate host defense stratagems.

It remains elusive how NLRs detect diverse and broad types of cellular perturbation. Similarly, it is unclear how NLRs adapt to different types of cellular dysfunction. It has been postulated that NLRs could maintain inactive states by means of host decoy or guard proteins and that modifications of these proteins induced by pathogens trigger the activation of NLRs [209]. In the same direction, it is reasonable to hypothesize that cellular homeostasis dysfunction could modify host proteins or their activities and thereby activate NLRs. Indeed, generation of S1P is increased upon perturbation of cellular homeostasis and subsequently S1P triggers NOD1/2-medidated activation of innate immunity [181]. Considering that cellular homeostasis disruption induced by pathogens converges toward conserved pathways and molecules, these altered proteins/metabolites are designated as SAMPs. Accordingly, NLRs could detect the perturbation of cellular homeostasis by monitoring SAMPs such as S1P. The identities of SAMPs activating other NLRs upon distinct types of cellular stress, how SAMPs are generated and the distinct mechanisms employed by NLRs to differentially detect SAMPs, PAMPs or DAMPs remain to be elucidated. Given that many SNPs in NLRs are associated with autoimmune diseases, the SAMPs–NLRs axis likely represents an evolutionary conserved mechanism involved in infection, sterile inflammation and autoimmune disorders. Comprehensive understanding of the mechanisms of NLRs activation will help to harness the power of NLRs to fight infections by boosting inflammatory responses or reducing excessive inflammation in NLRs-associated autoinflammatory disorders.

## Data Availability

Not applicable.
